# Effect of smoking and smoking cessation intervention on postoperative complications of orthopedic and hand surgery: A single-center interventional study

**DOI:** 10.18332/tpc/214133

**Published:** 2026-01-09

**Authors:** Antti Kyrö, Kalle Kainiemi, Antti Malmivaara, Sara Taskinen, Tiina Laatikainen

**Affiliations:** 1 Surgical Outpatient Department, Päijät-Häme Central Hospital, Wellbeing Services County of Päijät-Häme, Lahti, Finland; 2 Department of Acute Medicine, Päijät-Häme Central Hospital, Wellbeing Services County of Päijät-Häme, Lahti, Finland; 3 Finnish Institute for Health and Welfare, Helsinki, Finland; 4 Orton Orthopedic Hospital, Helsinki, Finland; 5 Department of Mathematics and Statistics, University of Jyväskylä, Jyväskylä, Finland; 6 Institute of Public Health and Clinical Nutrition, Faculty of Medicine, University of Eastern Finland, Kuopio, Finland; 7 Wellbeing Services County of North Karelia (Siun Sote), Joensuu, Finland

**Keywords:** smoking, smoking cessation intervention, orthopedics, hand surgery, complications

## Abstract

**INTRODUCTION:**

Smoking impairs the outcome of surgery for many reasons. This study aimed to determine the effect of smoking, smoking cessation intervention, and other factors on the incidence of postoperative complications after elective orthopedic and hand surgery directed at bone or cartilage attached at the bone.

**METHODS:**

Altogether 451 patients, aged 18 years, participated in this single-center interventional study in Finland between 2015 and 2019. Their smoking status was confirmed by laboratory tests. The study's outcome was the occurrence, number, and severity of complications. A complication was defined as a prolonged hospital stay or any additional treatment or visit to a healthcare unit within 12 months, and its severity was determined by a modified Clavien-Dindo-Sink (CDS) index.

**RESULTS:**

Nine out of 80 smokers stopped smoking with laboratory verification before surgery, and additionally, six smokers stopped smoking at or after surgery. In comparison to never smokers, continuing smokers had more often at least one complication (adjusted odds ratio, AOR=2.50; 95% confidence interval, CI: 1.23–5.09; p<0.05) and more complications (adjusted risk ratio, ARR=1.58; 95% CI: 1.08–2.30; p<0.05). Further, the odds of being in a higher CDS-index category compared to the lower category (AOR=2.19; 95% CI: 1.14–4.19; p<0.05) also increased. With decreasing age, the occurrence (AOR = 0.98; 95% CI: 0.96–1.00; p<0.05) and number of complications (ARR=0.98; 95% CI: 0.97–1.00; p<0.01) decreased. Additionally, the odds of being in a higher CDS-index category compared to a lower category increased (AOR=0.98; 95% CI: 0.96–1.00; p<0.05). In comparison to men, women were more likely to have at least one complication (AOR=1.68; 95% CI: 1.03–2.75; p<0.05). With increasing alcohol consumption, the occurrence of at least one complication increased (AOR=1.10; 95% CI: 1.02–1.19; p<0.05).

**CONCLUSIONS:**

Continuing to smoke and decreasing age, the occurrence, number, and severity of complications increased. Alcohol consumption and female sex were linked to an elevated occurrence of complications. It is important to wean every smoking patient, especially young smokers, from smoking to reduce the occurrence and severity of complications.

## INTRODUCTION

Smoking impairs the outcome of surgery for many reasons. It harmfully impairs blood circulation in the area of operation^1^, oxygenation of tissues^2^, and immune response to infections^3^. Further, it increases the risk of atherosclerosis^4^ and excessive surgical bleeding^5^. Smoking increases abnormal histopathological alterations in muscles^6^ and reduces muscle strength^7^.

It is important that in all contacts with healthcare personnel, smokers are informed about the benefits of smoking cessation. Especially at surgery, smoking patients are receptive to quitting smoking^8^ and so lower the risk of complications and achieve all other benefits of smoking cessation. Therefore, before and at the time of surgery, smoking cessation is more likely to be successful. Some harmful effects of smoking are reversible^9^. The longer the abstinence is, the greater the mean lowering effect on the rate of complications^10^.

In a study of hip or knee arthroplasty patients, smokers had an elevated risk (36%) of any complication compared with that of non-smokers (20%)^11^. The postoperative 90-day overall complication rate in orthopedic surgery was 29.9% for current smokers, 31.1% for ex-smokers, and 23.2% for never smokers. In adjusted logistic regression analysis of overall complication rate, current smokers had an odds ratio (OR) of 1.36 and ex-smokers of 1.01, correspondingly^12^. In a study of inpatient total hip arthroplasty patients, the overall complication rate for smokers was 9.60% and for non-smokers 4.31%^13^. The 30-day total complication rate in primary total hip or total knee arthroplasties was 5.9% for current smokers, 6.9% for ex-smokers, and 5.4% for non-smokers^14^. In multivariate analysis of overall complication rate, current smokers and ex-smokers had a higher OR of 1.18 and 1.20, respectively, compared with that of non-smokers^14^.

In a study of inpatient total hip arthroplasty patients using an adjusted multivariable logistic model, smokers had a higher OR for myocardial infarction, cardiac arrest, deep vein thrombosis, pneumonia, urinary tract infection, sepsis, acute renal failure, discharge to a skilled nursing facility, and mortality^13^.

In an effective smoking cessation intervention study of hip or knee replacement patients, the overall complication rate of the study patients within 4 weeks was 18% in the intervention group and 52% in the control group, with a relative risk of 0.34^15^. In another effective smoking cessation intervention study of orthopedic or general surgery patients, the overall complication rate in 30 days was 21%, (41% in controls), with a relative risk of 0.51^16^. Using effective intervention, 36% (and 2% of controls) were not smoking at least 3 weeks before surgery and until at least 4 weeks after surgery. After one year, 33% of the intervention group (and 15% of controls) were not smoking^17^. In a study of posterior instrumented fusion, patients who quit smoking after surgery for longer than 6 months had a 17% non-union rate; however, patients who continued smoking after surgery had a 26.5% non-union rate^18^.

The aim of this study was to determine the effect of smoking and smoking cessation intervention on the incidence of postoperative complications after orthopedic and hand surgery. Additional aims were to define the feasibility of a preoperative smoking cessation intervention and its effect on reducing smoking, and to determine which other factors other than smoking, such as chronic diseases, influence the occurrence of complications^19^.

## METHODS

### Study design and patients

This single-center interventional study was conducted in Päijät-Häme Central Hospital, Finland. The patients included in the study had been put on a waiting list for orthopedic or hand surgery, mostly in the outpatient department, and were recruited between October 2015 and April 2018. The criteria for eligibility were age ≥18 years and that the planned surgery was directed at the bone or cartilage attached to the bone. Patients who had had a malunited or non-united fracture for at least two months were eligible for the study. Patients with acute fractures and patients put on the waiting list for other types of surgery were excluded.

Patients were classified as smokers or non-smokers according to self-reports, which were corrected if laboratory tests for cotinine in urine (U-Cot) and carboxyhemoglobin (Hb-CO) showed a recent history of smoking. The smoking history of the study patients was classified according to the Finnish National Institute for Health and Welfare (THL) survey classification: 1=daily smoker, 2=occasional smoker, 3=quit smoking 1–12 months ago, 4=quit smoking >12 months ago, and 5=never smoker^20^.

The smoking status of the study patients was defined using the following criteria. A participant was a smoker if that person reported daily smoking, and/or U-Cot was >250 ug/L, and Hb-CO was above 2%. A participant reporting exposure to passive smoking and/or whose U-Cot was 100–250 ug/L was a passive smoker. Non-smokers showed U-Cot <50 ug/L and Hb-CO <2%^19^. Patients using only snuff or other smokeless nicotine products who had Hb-CO values <2% but elevated U-Cot values were classified as non-smokers.

The study protocol has been previously published^19^ and registered in the ISRCTN registry (ID 16961081). Baseline and follow-up data were collected using questionnaires, phone calls, laboratory tests, and information from patient records. All participants filled in a baseline questionnaire. At 3, 6, and 12 months after surgery, patients were interviewed by phone or answered a short questionnaire sent by post. In addition, data from patient records were collected for the whole follow-up period. Smoking patients received written and verbal information about the health and economic advantages of smoking cessation and were encouraged to stop smoking.

During the pre-operative assessment, at about two weeks before the operation, the laboratory tests, U-Cot and Hb-CO, were taken to verify the smoking habit and to differentiate the use of other nicotine products. Post-operatively, Hb-CO and U-Cot tests were repeated to verify smoking or non-smoking habits^19^.

The patient’s age was the age in years on the day of the operation. The study patient was a woman if the individual number, i.e. the remainder of the personal identification number, was even and a man if the individual number was odd. The length of education was the number of years the patient studied in school. Body mass index (BMI) was obtained by dividing a patient’s weight (kg) by height squared (m^2^).

The Charlson Comorbidity Index (CCI) was used to study the effect of patient comorbidities on the occurrence of complications^21^. CCI can be used without the influence of age, i.e. CCI excluding age^22^. In our study, CCI excluding age is used, as the age of the patients is another independent factor that predicts complications. The CCI index excluding age is the sum of the diseases of the patient. Simplifying, myocardial infarct, congestive heart failure, peripheral vascular disease, cerebrovascular disease, dementia, chronic pulmonary disease, rheumatic disease, peptic ulcer disease, mild liver disease, and uncomplicated diabetes each yield one point. Hemiplegia or paraplegia, diabetes with chronic complications, renal disease, and any malignancy each yield two points. Moderate or severe liver disease yields three points, and metastatic solid tumor and AIDS yield six points. These possible points of the patient are summed. Patients had cancer if they had an ICD-10 diagnosis of C00-C97, systemic inflammatory disease with arthritis if they had an ICD-10 diagnosis M05-M14, M31.5, M32-M34, M35.1 or M35.3, and diabetes if they had an ICD-10 diagnosis E10-E14.

The outcome of the study was the absence or occurrence, number, and severity of complications. A complication was defined as follows: any event causing additional medical or surgical treatment, an additional radiological or laboratory test, a prolonged stay in hospital, or any additional visit to a health care unit^23^. A prolonged stay in hospital usually meant about double the treatment time compared with what a planned surgery usually required. It was decided on a case-by-case basis, and the decision was usually self-evident.

The description and ICD-10 codes for complications or reasons to contact the hospital or healthcare unit were recorded in the study form. After follow-up, the ICD-10 codes of complications or reasons for prolonged stay in hospital were checked, corrected if necessary, and added if they were missing from the patient’s study form.

We did not find a classification completely suitable to determine the severity of complications in our study; therefore, we slightly modified and used the Clavien-Dindo-Sink classification of complications (CDS-index)^24^. After determining the ICD codes, this CDS-index was determined with Grades from I to V to describe the severity of complications. In this study, Grade I includes 1 or 2 additional visits to a healthcare unit or prolonged stay in hospital, and extra laboratory tests, if needed, and prescribing of analgesics or antiemetics. Grade II includes at least three additional visits to a healthcare unit and harmful and prolonged symptoms needing antibiotics or other special treatment. Grade III includes treatable complications needing surgical or radiographic intervention or long ultrasound treatment to promote bone union. Grade IV includes a complication that is life-threatening, requires intensive care unit admission, or is not treatable, or has potential for permanent disability. Grade V means death connected with the surgery or with a complication of that surgery. Grades IV and V were combined because of the small number of study patients with these grades.

Information on patients’ characteristics such as age and gender, height and weight, or BMI, as well as medication and permanent diagnoses, to determine whether they had chronic conditions that might affect surgical outcomes was collected from patient records. Behavioral factors and socioeconomic indicators, including smoking status, weekly alcohol consumption, physical activity habits, nicotine dependence, and years of education, were collected using a questionnaire completed by the patients at baseline.

Participation in the study was voluntary. Patients willing to participate were given a detailed information brochure, and they signed an informed consent form. Ethical approval for the study was received from the Regional Ethical Committee of Tampere University Hospital, Finland (Approval number: R15129; Date 22 September 2015). Permission for the study was granted by the chief medical officer of Päijät-Häme Central Hospital, Lahti, Finland.

### Statistical analysis

The plan was to recruit a total of 550 patients for the study, aiming to have at least 20% of them daily smokers, corresponding approximately to the current smoking prevalence in Finland. The assumption was that as many as half of the smokers could quit smoking^19^. To test differences in means and proportions, we used analysis of variance (ANOVA) and chi-squared goodness-of-fit tests, respectively.

The main analyses were conducted using three statistical models: a logistic regression model to examine the occurrence of at least one complication, a Poisson regression model to assess the number of complications, and a proportional odds model to evaluate the severity of complications^25^. For each outcome variable, the fixed effects included smoking status, age (on the day of surgery), gender, total physical activity, alcohol consumption, years of education, CCI excluding age, and body mass index (BMI). All analyses were performed using R^26^. The proportional odds model was fitted using the MASS package^27^. We consider a p<0.05 as evidence of statistical significance.

## RESULTS

### Patient characteristics

**Figure 1:**
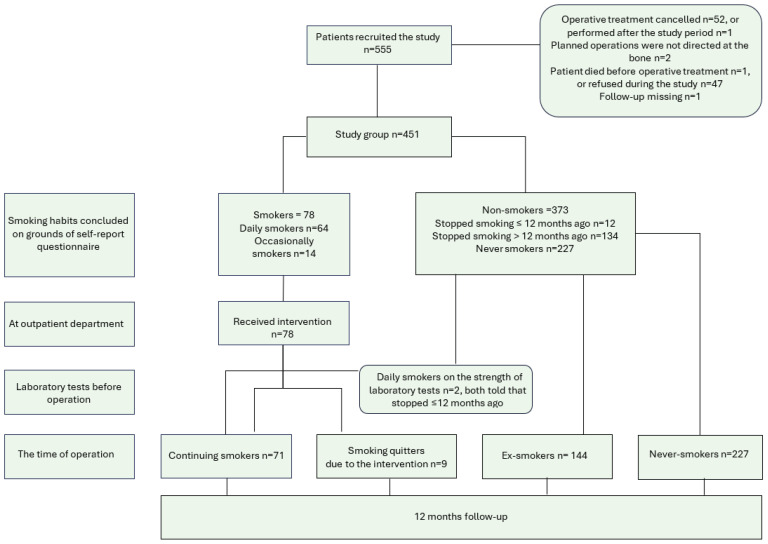
Flow chart of selection of orthopedic and hand surgery patients in the study on smoking cessation intervention conducted in 2015–2019 at Päijät-Häme Central Hospital

A total of 550 patients were recruited. In the initial phase of the recruitment period, it was noticed that there were not enough smokers, and in the end phase, recruitment was targeted especially at smokers. A total of 99 patients were lost in different phases of the study (Figure 1), so the final study group consisted of 451 patients: 280 women, 171 men, with a mean age of 63.8 years (Table 1). Out of 371 non-smokers, 240 were women. The mean age of smokers was 52.8 years, and that of non-smokers was 66.2 years (mean difference, MD=13.43; 95% CI: 10.26–16.60; p<0.001). Non-smokers on average used less alcohol (MD=1.43; 95% CI: 0.55–2.30; p<0.05), had on average more comorbidities on grounds of CCI excluding age (MD=0.35; 95% CI: 0.17–0.53; p<0.001), and had more systemic inflammatory diseases with arthritis (p<0.05) than smokers (Table 1). There was no significant difference in total physical activity, length of education, or BMI, nor in the occurrence of cancer (Table 1), diabetes, use of methotrexate, or use of cortisone between smokers and non-smokers.

**Table 1:** Orthopedic and hand surgery patients’ characteristics at the time of recruitment in the smoking cessation intervention conducted in 2015–2019 at Päijät-Häme Central Hospital (N=451)

*Characteristics*	*Smokers ^a^* *n (%)*	*Non-smokers* *n (%)*	*All* *n (%)*	*p*
**Total**	80 (17.7)	371 (82.3)	451 (100)	
**Age^b^** (years), mean (SD^c^)	52.77 (13.34)	66.20 (11.08)	63.82 (12.59)	<0.001
Women	40 (50.0)	240 (64.7)	280 (62.1)	0.020
Men	40 (50.0)	131 (35.3)	171 (37.9)	0.020
**Total physical activity** (min/week), mean (SD)	262.12 (272.52)	316.90 (349.13)	306.10 (337.08)	0.135
**Alcohol portions^d^ per week**, mean (SD)	2.90 (3.63)	1.48 (2.75)	1.73 (2.98)	0.002
**Education** (years)^e^, mean (SD)	11.95 (3.06)	11.90 (3.80)	11.91 (3.68)	0.912
**CCI excluding age^f^**, mean (SD)	0.40 (0.62)	0.75 (1.08)	0.69 (1.02)	<0.001
**BMI^g^**, mean (SD)	27.66 (5.45)	28.55 (4.97)	28.39 (5.06)	0.185
**Patients with cancer^h^**	6 (7.5)	50 (13.5)	56 (12.4)	0.199
**Patients with systemic inflammatory diseases with arthritis**	5 (6.3)	57 (15.4)	62 (13.7)	0.049

a Smoker: A patient reporting smoking and/or laboratory tests showing smoking.

b Age: age of the patient at the day of operation.

c SD: standard deviation.

d Alcohol portion: 12 g alcohol.

e Education (years): number of years in school and studied after school.

f CCI excluding age: Charlson Comorbidity Index without the influence of age, range 0–7 in our study patients.

g BMI: body mass index (kg/m^2^).

h Patient with cancer with an ICD-10 diagnosis of C00-C97.

i Patient with systemic inflammatory disease with arthritis with an ICD-10 diagnosis of M05-M14, M31.5, M32-M34, M35.1 or M35.3.

Never smokers were on average the oldest and continuing smokers the youngest (F-test, F=32.14; df1=3, df2=449; p<0.001). Female patients predominated among the never smokers. Never smokers used the least alcohol, and continuing smokers used it the most (F=9.23; df1=3, df2=422; p<0.001). Never smokers seemed to have insignificantly more physical activity on average than smokers or ex-smokers (Table 2).

**Table 2:** Orthopedic and hand surgery patients’ characteristics in the smoking cessation intervention conducted in 2015–2019 at Päijät-Häme Central Hospital, by confirmed smoking status before surgery (N=451)

*Characteristics*	*Continuing* *smokers ^a^* *n (%)*	*Smoking* *quitters ^b^* *n (%)*	*Ex-smokers* *n (%)*	*Never smokers* *n (%)*	*All* *n (%)*	*p*
**Total**	71 (15.7)	9 (2.0)	144 (31.9)	227 (50.3)	451 (100)	
**Age** (years)^c^, mean (SD^d^)	52.65 (12.97)	53.77 (16.84)	64.06 (10.31)	67.56 (11.35)	63.82 (12.59)	<0.001
Women	35 (49.3)	5 (55.6)	71 (49.3)	169 (74.4)	280 (62.1)	0.010
Men	36 (50.7)	4 (44.4)	73 (50.7)	58 (25.6)	171 (37.9)	<0.001
**Occasional smokers/daily smokers**	12/59	2 / 7				
	***Mean (SD)***	***Mean (SD)***	***Mean (SD)***	***Mean (SD)***	***Mean (SD)***	
**Fagerström test at recruitment**	1.76 (1.41)	1.17 (1.33)	0.01 (0.08)	0 (0)	0.29 (0.86)	<0.001
**Total physical activity** (min/week)	260.04 (265.81)	277.61 (336.23)	262.10 (223.75)	351.27 (406.27)	307.10 (337.08)	0.055
**Alcohol portions^e^ per week**	2.93 (3.78)	2.71 (2.31)	2.20 (3.71)	1.01 (1.76)	1.73 (2.98)	<0.001
**Education** (years)^f^	11.82 (3.01)	12.89 (3.44)	11.92 (4.00)	11.89 (3.68)	11.91 (3.68)	0.878
**CCI excluding age^g^**	0.39 (0.64)	0.44 (0.53)	0.81 (1.05)	0.70 (1.10)	0.68 (1.02)	0.035
**BMI^h^**	27.72 (5.55)	27.25 (4.87)	29.00 (5.04)	28.26 (4.91)	28.39 (5.06)	0.268

a Continuing smoker: patient who continued smoking despite intervention.

b Smoking quitter: patient who quit smoking by intervention.

c Age (years): age of the patient on the day of surgery.

d SD: standard deviation.

e Alcohol portion: 12 g alcohol.

f Education (years): number of years in school and studying after school.

g CCI excluding age: Charlson Comorbidity Index without the influence of age, range 0–7 in our study patients.

h BMI: body mass index (kg/m^2^).

### Success of smoking cessation intervention

Nine (11.3%) out of 80 smoking patients stopped smoking before surgery and showed normal Hb-CO and U-Cot values. Of these nine patients, seven stopped smoking on average 3.1 months (range: 1–6) before surgery.

Additionally, six patients (7.5%) stopped smoking at the time of or after the surgery. Most of them quit at the time of surgery or within 1 month of surgery and showed normal Hb-CO and U-Cot values later, during the follow-up period.

A total of 16 (20.0%) smoking patients disclosed that they had reduced the number of cigarettes smoked per day or per week. The calculated, known mean reduction in the number of cigarettes smoked was 40%. Thus, in the follow-up period, the patients with known reduction smoked on average 60% of the number of cigarettes smoked initially.

### The occurrence, number, and severity of complications in four patient groups by smoking habit

A total of 280 out of 451 patients (62.1%) had no complications. Two-thirds of never smokers, 157/227 (69.2%), had no complications, and of those who continued smoking, 41/71 (57.7%) had at least one complication. Continuing smokers had on average 0.99 complications and never smokers correspondingly 0.55 complications (p=0.006) (Table 3).

**Table 3:** Number of orthopedic and hand surgery patients with or without complications, mean number and severity of complications among patients in the smoking cessation intervention conducted in 2015–2019 at Päijät-Häme Central Hospital, by confirmed smoking status before surgery (N=451)

*Number*	*Continuing* *smokers ^a^* *n (%)*	*Smoking* *quitters ^b^* *n (%)*	*Ex-smokers* *n (%)*	*Never smokers* *n (%)*	*All* *n (%)*	*p*
**Total**	71 (15.7)	9 (2.0)	144 (31.9)	227 (50.3)	451 (100)	
**Patients with complications ≥1**	41 (57.7)	5 (55.5)	55 (38.2)	70 (30.8)	171 (37.9)	0.011
**Patients with no complications**	30 (42.3)	4 (44.4)	89 (61.8)	157 (69.2)	280 (62.1)	0.079
	***Mean (SD) ^c^***	***Mean (SD)***	***Mean (SD)***	***Mean (SD)***	***Mean (SD)***	
**Mean number of complications**						
All	0.99 (1.11)	0.67 (0.71)	0.58 (0.83)	0.55 (0.94)	0.63 (0.94)	0.006
Women	1.00 (1.06)	1.00 (0.71)	0.69 (0.84)	0.55 (0.92)	0.65 (0.93)	0.050
Men	0.97 (1.18)	0.25 (0.50)	0.47 (0.82)	0.53 (1.00)	0.60 (0.98)	0.058
**Patients with cancer^d^**	0.40 (0.55)	0 (0)	0.68 (0.89)	0.55 (1.09)	0.57 (0.97)	0.862
**Patients with systemic inflammatory diseases with arthritis^e^**	0.75 (0.96)	0 (0)	0.58 (0.86)	0.65 (1.11)	0.61 (0.98)	0.915
**Patients with diabetes^f^**	0 (0)	0 (0)	0.71 (1.01)	0.62 (0.90)	0.61 (0.92)	0.369
**Severity of the complications**						
**CDS index^g^**	1.15 (1.29)	1.22 (1.30)	0.72 (1.06)	0.60 (1.06)	0.74 (1.12)	0.002

a Continuing smoker: patient who continued smoking despite intervention.

b Smoking quitter: patient who quit smoking by intervention.

c SD: standard deviation.

d Patient with cancer with an ICD-10 diagnosis of C00-C97.

e Patient with systemic inflammatory disease with arthritis with an ICD-10 diagnosis of M05-M14, M31.5, M32-M34, M35.1 or M35.3.

f Patient with diabetes with an ICD-10 diagnosis E10-E14.

g CDS-index: modified Clavien-Dindo-Sink classification of severity of the complication (0–5).

Continuing smokers had more often at least one complication (AOR=2.50; 95% confidence interval, CI: 1.23–5.09; p<0.05) and more complications (adjusted risk ratio, ARR=1.583; 95% CI:1.08–2.30; p<0.05) than never smokers. Also, the odds of being in a higher CDS-index category compared with a lower category increased (AOR=2.19; 95% CI: 1.14–4.19; p<0.05) (Table 4). Never smokers had, on average, a lower mean severity of complications than the patients of other smoking categories (p=0.002) (Table 3). Six patients (1.3%) out of 451 died during the follow-up period for reasons connected or unconnected with the surgery.

**Table 4:** Factors influencing the occurrence, number and severity of complications among orthopedic and hand surgery patients in the smoking cessation intervention conducted in 2015–2019 at Päijät-Häme Central Hospital, by confirmed smoking status before surgery (N=451)

*Variables*	*Occurrence of at least one* *complication*		*Number of complications*		*Severity of complications* *CDS-index^a^*	
	*AOR*	*95% CI*	*ARR*	*95% CI*	*AOR*	*95 % CI*
**Smoking**						
Never smokers (ref.)						
Ex-smokers	1.290	0.773–2.149	1.063	0.775–1.455	1.257	0.764–2.063
Smoking quitters^b^	1.983	0.630–6.217	1.077	0.501–2.037	2.029	0.676–5.759
Continuing smokers^c^	2.500**	1.234–5.087	1.583**	1.078–2.300	2.185**	1.135–4.191
**Age** (years)^d^	0.977**	0.958–0.997	0.984***	0.973–0.995	0.980**	0.961–0.998
**Gender**						
Male (ref.)						
Female	1.675**	1.034–2.748	1.256	0.945–1.681	1.439	0.907–2.304
**Total physical activity** (hours/week)	1.030	0.992–1.069	1.018	0.996–1.038	1.021	0.986–1.056
**Alcohol portions^e^ per week**	1.097**	1.016–1.190	1.016	0.972–1.057	1.051	0.984–1.122
**Education** (years)^f^	0.945*	0.886–1.005	0.972	0.936–1.009	0.957	0.901–1.013
**CCI excluding age^g^**	1.067	0.854–1.327	1.046	0.913–1.186	1.081	0.876–1.322
**BMI^h^**	1.003	0.962–1.045	1.006	0.982–1.030	0.999	0.959–1.039

ARR: adjusted risk ratios when the number of complications was modelled using the Poisson regression model. AOR: adjusted odds ratios when the severity of complications (CDS-index) was modelled using the proportional odds model, or when the occurrence of at least one complication was modelled using the logistic regression model.

a CDSindex: modified Clavien-Dindo-Sink classification of severity of the complication (0–5).

b Smoking quitter: patient who quit smoking by intervention.

c Continuing smoker: patient who continued smoking despite intervention.

d Age (years): age of the patient at the day of operation.

e Alcohol portion: 12 g alcohol.

f Education (years): number of years in school and studying after school.

g CCI excluding age: Charlson Comorbidity Index without the influence of age, range 0-7 in our study patients.

h BMI: body mass index (kg/m^2^).

* p<0.10,

** p<0.05,

*** p<0.01.

### Other factors influencing the occurrence, number, and severity of complications

With decreasing age by one year, the occurrence of patients with at least one complication (AOR=0.98; 95% CI: 0.96–1.00; p<0.05) and the mean number (ARR=0.98; 95% CI: 0.97–1.00; p<0.01) of complications increased. In addition, the odds of being in a higher CDS-index category compared to a lower category increased (AOR=0.98; 95% CI: 0.96–1.00; p<0.05). Female patients more often had at least one complication than male patients (AOR=1.68; 95% CI: 1.03–2.75; p<0.05). With increasing alcohol consumption by one portion per week, the number of patients with at least one complication increased (AOR=1.10; 95% CI: 1.02–1.19; p<0.05). With one year longer time in education, the occurrence of patients with at least one complication seemed to decrease (AOR=0.95; 95% CI: 0.89–1.01; p<0.10). Neither CCI (range 0–7 in our study), nor BMI influenced the occurrence or severity of complications (Table 4).

### The effects of smoking cessation by the intervention

Those who had quit smoking had, on average, 0.67 complications, and separately male quitters had 0.25 complications, and seemed to have, on average, fewer complications than continuing smokers and male continuing smokers, who had 0.99 and 0.97 complications, respectively (Table 3). The differences were not significant. The occurrence, number, and severity of complications of quitters did not differ significantly from the corresponding values of never smokers. However, the number of quitters was small (Table 4).

## DISCUSSION

In our study, non-smokers were older, used less alcohol, had more comorbidities, and had systemic inflammatory diseases with arthritis more often than smokers. Both by continuing to smoke and at a younger age, the occurrence, number, and severity of complications increased. With increasing alcohol consumption, and also in women compared to men, there was an increased risk of getting at least one complication. Ex-smokers did not have a statistically significantly higher occurrence, number, or severity of complications than never smokers; it seems that this is because of smoking cessation.

Møller et al.^15^ carried out smoking cessation interventions, including weekly counseling with a trained nurse and offering patients free nicotine replacement therapy (NRT), and initiated these 6–8 weeks before hip or knee replacement. The overall complication rate of the study patients within 4 weeks was 0.18 in the intervention group and 0.52 in the control group, with a relative risk of 0.34^15^. In our study, the complication rate within one year after surgery was higher, 0.99 for continuing smokers and 0.67 for those who quit smoking due to the intervention. However, our smoking cessation intervention was less effective, the follow-up time was much longer, and the definition of complications was more inclusive, for instance, including pain reported by the patient.

In a study by Gräsbeck et al.^12^, the rate of Clavien-Dindo grade IV-V complication, i.e. life-threatening complication and death, occurred after all surgeries in 5.2% of current smokers, in 6.8% of ex-smokers, and in 3.3% of never smokers. In adjusted logistic regression analysis of Clavien-Dindo grade IV-V complication rate^12^, current smokers had an odds ratio of 1.36 and ex-smokers 1.01. In line with these findings, in our study, continuing smokers had, on average, more severe complications than never smokers.

Smokers were younger than non-smokers in our study, as found in earlier studies^13,18^. The increasing age of the patient is associated positively with the revision rate of total hip arthroplasty^28^ and the incidence of deep-vein thrombosis after foot and ankle surgery^29^. However, increasing age associates positively with patient satisfaction after total joint replacement^30^. In our study, with increasing age of the patient, the occurrence of at least one complication, the number, and the severity of complications decreased. This is contrary to what is generally found in the literature. We suppose that younger patients presume a better outcome of surgery, but older patients are satisfied with a worse outcome without seeking health care.

Women patients in our study had surprisingly more complications than men, which is contrary to what is generally found earlier^18^. Especially, female smoking quitters and ex-smokers in our study seemed to have more complications than the corresponding men did. Among never smokers, there was no difference between women and men. Maybe smoking is more harmful for women than for men, as found earlier^31^. Smokers engaged in less physical activity and used more alcohol than non-smokers. Higher physical activity has been associated with better pain relief and better subjective outcomes after orthopedic surgery^32^. Increased alcohol consumption is associated with increased risk of postoperative complications^33^. In these respects, non-smokers had healthier lifestyles and possibilities to further lower complication rates.

Higher education predicts better leg pain relief and better outcomes after spine surgery^34^. Similarly, in our study, higher education seemed to reduce the occurrence of at least one complication.

Not all patients were willing to participate in this study, as participants were required to fill in a long questionnaire, to comply with extra laboratory tests taken twice, and to answer follow-up phone calls or a letter three times. Smoking patients are not usually eager to participate in studies of smoking behavior^35^. Warner et al.^35^ found that only 10% of patients with eligibility criteria provided consent to their study. As smoking is increasingly unacceptable and restricted, it is possible that some of the patients experienced social pressure because of their smoking habit and, therefore, refused to participate or continue in the study.

Only nine of our 80 smoking patients stopped smoking, verified by laboratory tests before surgery. This shows that we did not succeed well enough in the smoking cessation intervention, unlike in an earlier study^15^. We performed the research mainly in addition to our own daily work. Free NRT seems to increase^15^ the rates of smoking cessation. In our study, we could not provide free NRT or any other actual incentives.

### Strengths and limitations

The strengths of our study include that the study group was recruited from a central hospital serving the whole population of a catchment area. Further, we had laboratory confirmation of smoking or non-smoking and a long (12 months) follow-up of patients. The definition of complications was clear, including a prolonged stay in hospital or any additional postoperative contact with health care. Our study group was uniform: it included patients with planned orthopedic or hand surgery directed at bone or cartilage attached to the bone. The number of patients recruited is relatively large. We could show the effect of smoking and age on the occurrence, number, and severity of postoperative complications. We also showed the effect of gender and alcohol consumption on the occurrence of complications.

Our study has limitations. Despite previous evidence of the effectiveness of smoking cessation, a randomized trial design was not feasible. Our study has potential selection bias. We were unable to recruit enough smokers or smokers who could quit smoking. There are potential confounding factors in the study. We did not regard detailed information about the surgery, such as the surgeon performing the operation, the length or cleanliness class of surgery, or the ASA class of the patient. The researchers mainly conducted the study in addition to their own patient work at the non-teaching central hospital. This had the advantage of producing the intervention and outcomes in real-world circumstances. However, from a research point of view, the study has been challenging, for example, in recruiting patients. The number of smokers and especially quitters recruited for our study was small. Due to the small number of quitters in our study, the effect of smoking cessation on the occurrence and severity of postoperative complications could not be shown. Furthermore, our smoking cessation intervention was not as effective as we anticipated. We believe that our results can be generalized to some extent to other hospitals and countries, as the procedures are performed in a broadly similar manner worldwide. However, with better organization and planning, maybe many more smokers and quitters could be recruited for the study.

## CONCLUSIONS

Our study confirmed that continued smoking is associated with a higher risk, number, and severity of postoperative complications compared with never smokers. Patients who quit smoking, although few in number, experienced complication rates comparable to never smokers, suggesting clear benefits of cessation. However, the intervention used in this study was only modestly effective, as relatively few patients quit smoking before surgery. In addition, younger age, female sex, and higher alcohol consumption were also linked to elevated occurrence of complications, highlighting the multifactorial nature of postoperative risk. Younger age was also associated with a higher number and severity of complications. Our findings support the importance of smoking cessation in all, especially in younger, surgical patients, to reduce the occurrence and severity of complications. However, additional and more extensive studies are needed to provide sufficient evidence of the benefits of smoking cessation before orthopedic and hand surgery.

## Data Availability

The data supporting this research cannot be made available for privacy or other reasons.
